# Supplementary material for: Assessing the Current Integration of Multiple Personalised Wearable Sensors for Environment and Health Monitoring
Source: Sensors (Basel). 2021 Nov 19;21(22):7693. doi: 10.3390/s21227693 (PMC8620646; doi:10.3390/s21227693)
Supplement: Supplementary file 1 [file sensors-21-07693-s001.zip › sensors-1446638-supplementary.pdf]

Table S1. Sensors and data of the reviewed studies.

No	Reference	location (L), Time(T), Setting (S), Design (D)	Sample Size (N), Age Restriction Structure (A), Gender (G)	Environment Domains (ED), Sensors	Environment -Related Outcome Variables	Health Domains (HD), Sensors	Health-Related Outcome Variables	Statistical Analysis	Supplementary Data
1	Benita, et al. [1]	L = Singapore; T = 10 minutes; S = 700 m walking route.	N = 10; A = 21–25; G = Female.	ED = Physical Environment; Sensor 1: Kestrel 5400; Sensor 2: Smartphone.	Data 1: air temperature, relative humidity, wind speed and mean radiant temperature; Data 2: environmental noise; Contextual: POI data.	HD = Activity and Mental health; Sensor 3: Empatica 4; Sensor 2: Smartphone.	Data 3: skin temperature and Electrodermal activity (EDA); Data 2: GPS and speed.	1) Descriptive statistics; 2) classifier algorithms are MNL, RFC and SVM; 3) Geo-visualization;	1) Pre- and post-questionnaires
2	Benita and Tunçer [2]	L = Singapore; T = 10 minutes; S = 700 m walking route.	N = 10; A = 21/25; G = Female.	ED = Physical and urban Environment; Sensor 1: Kestrel 5400; Sensor 2: Smartphone.	Data 1: Air temperature, relative humidity, wind speed and atmospheric pressure; Data 2: environmental noise. Contextual: Sky exposure in the city: 49 photographs were taken within about 10-15m distance;	HD = Activity and Mental health; Sensor 3: Empatica 4; Sensor 2: Smartphone	Data 3: Body skin temperature and Electrodermal activity (EDA); Data 2: Geo-coordinates, and walking speed	1) Hierarchical clustering; 2) Stepwise regression, Ridge regression (RR), Lasso regression (Lasso) and Random Forest (RF); 3) Pattern recognition tools and parametric tests to identify stress hotpots	1) Pro-Survey: Personal characteristics; 2) Post-survey: perceived Restorativeness : Hartig's Short-version Revised Perceived Restorativeness Scale (SRPRS) and Perceived Stress Scale (PSS))

3	Birenboim, et al. [3]	L = Utrecht , Netherland;<br>T = 30 minutes;<br>S = 3 km walking route.	N = 15(12);<br>A = 21.8;<br>G = Male.	ED = Urban Environment;<br><br>No particular sensor Geographic information system (GIS) environment.	Subjective data: survey to environment: Complete a questionnaire to rank (¨1¨ most relaxing and ¨8¨ most stressful) Contextual: Predefined characteristics of the walking route segments	HD = Mental health;<br><br>Sensor 1: GPS receiver;<br>Sensor 2: Microsoft Band (MS Band);<br>Sensor 3: Empatica E4.	Data 1: Geo-coordinates;<br>Data 2: Distance traveled, elevation, number of steps;<br>Data 3: Heart Rate (HR), EDA, skin temperature.	1) Geo-visualization ;	1) A third-party Android mobile application ¨Data Log¨ to log the measurements for Microsoft Band
4	Bohmer, et al. [4]	L = Rotterdam, Netherlands;<br>T = 7–10 days<br>S = Naturalistic.	N = 82(48);<br>A = 50 years or older;<br>G = Both.	ED = Physical Environment;<br><br>Sensor 1: light sensor.	Data 1:<br>Light exposure.	HD = Activity;<br><br>Sensor 2: accelerometer.	Data 2: Walk speed, bedtime.	1) Descriptive statistics;	1) Professional caregivers filled out questionnaires on the activities of daily living (ADL) and mobility of the participant.
5	Boissy, et al. [5]	L = Montreal, Canada;<br>T = 14 days;<br>S = Naturalistic.	N = 75(54);<br>A = 55–85 years of older;<br>G = Both.	ED= Urban Environment;<br><br>No particular sensor.	Qualitative data: subjective survey about the proximity to neighbour services.	HD = Activity;<br><br>Sensor 1: GPS receiver;<br>Sensor 2: A 3-axis accelerometer.	Data 1:<br>Geo-coordinates;<br>Data 2:<br>Acceleration.	1) Descriptive statistics;<br><br>2) detection algorithm.	1) A performance evaluation and self-report questionnaire
6	Bolliger, et al. [6]	L = Ghent, Belgium;<br>T = 15 days;<br>S = Naturalistic;	N = 5 (real-world);<br>A = Adult;<br>G = Both.	ED = Social Environment;<br><br>Sensor 1: Smartphone sensor.	Data 1: Applications, Barometer, Bluetooth, Light, Location, GPS, Screen, Temperature and voice activity, etc.	HD= Mental health and psychology;<br><br>Sensor 2: Empatica wristband;	Data 2: Physiological Data;<br>Data 1: Ecological Momentary Assessment.	No particular analysis.	1) Online survey (demographics, work- and health-related information);

						Sensor 1: Smartphone with the STRAW App.			2) blood pressure and heart rate measurement during briefing; 3) personalized feedback report based on their own study results
7	Borghi, et al. [7]	L = Milan, Italy; T = 14 days (repeat in two seasons); S = Predefined (90km home-to-work route);	N = 1: A = Adult; G = Not mentioned.	ED = Physical Environment; Sensor 1: DiSCmini; Sensor 2: $PM_{2.5}$ monitor; Sensor 3: CairClip NO2.	Data 1: UFP exposure; Data 2: $PM_{2.5}$ concentrations; Data 3: NO2 concentrations.	HD = Physical health; Sensor 4: Heart rate monitor.	Data 4: Heart rate and geo-coordinates.	1) Descriptive Analysis	
8	Burgi, et al. [8]	L = Zurich, Switzerland; T = A week; S = Naturalistic;	N = 123(119); A = 11–14; G = Both.	ED= urban environment; No particular sensor	Contextual: Geospatial data (land use data, street network and points-of-interest);	HD= Activity; Sensor 1: A GPS receiver; Sensor 2: Accelerometer	Data 1: Geo-coordinates; Data 2: short, intermittent bursts of activity.	1) Descriptive statistics	1) Complete a diary;
9	Butt, et al. [9]	L = Cambridge, USA; T = Two weeks; S = Naturalistic;	N = 20 (11); A = 24/35; G = Both.	ED = Social environment; Sensor 1: Smartphone augmented with ¨FunF	Data 1: Sensed and recorded proximity to nearby phones, implying people nearby.	HD = Activity; Sensor 2: Wireless system (ZEO).	Data 2: Total sleep time (TST), rapid eye movement (REM), time in non-slow-wave NREM (light NREM), sleep and	1) Spearman's rank correlations; 2) Principal component analysis; 3) Wilcoxon sign-ranked test.	

				sensing platform¨.			wake time, time taken to fall asleep.		
10	Cerin, et al. [10]	L = Texas, USA;<br>T = A week;<br>S = Naturalistic;	N = 84 (73/66);<br>A = 3/5children and their parents;<br>G = Both.	ED = urban environment;<br><br>No particular sensor.	Qualitative data: Perceived traffic safety, traffic hazards, Perceived signs of physical and social disorder.	HD = Activity;<br><br>Sensor 1: GPS receiver;<br>Sensor 2: Accelerometers.	Data 1: Geo-coordinates;<br>Data 2: Physical activity (PA) and reducing sedentary behavior (SB).	1) Generalized additive mixed models (GAMMs).	1) Parents completed a survey:
11	Chaix, et al. [11]	L = Paris, France;<br>T = A week;<br>S = Naturalistic;	N = 319(285);<br>A = Average 50.2;<br>G = Both.	ED = urban environment;<br><br>No particular sensor.	Qualitative data: Questionnaire assessment of transport (Studies of residential characteristics and qualitative outcomes on mode choice).	HD = Activity;<br><br>Sensor 1: GPS receiver, TripBuilder Web mapping application;<br>Sensor 2: Accelerometers.	Data 1: Geo-coordinates;<br>Data 2: Physical activity (PA).<br>Activity data: Phone-administered web mobility survey.	1) Descriptive statistics	
12	Chrisinger and King [12]	L = San Francisco, USA;<br>T = 20–25 mins;<br>S = Walking route;	N = 14;<br>A = -<br>G = Both.	ED= Social and urban environment;<br><br>Sensor 1: Smartphone/tablet-based application, Discovery Tool (DT).	Data 1: Photos and audio narratives about elements of the built environment that contributed to or detracted from their well-being.	HD = Mental health;<br><br>Sensor 1: Smartphone based GPS;<br>Sensor 2: Empatica E4.	Data 1: Geo-coordinates;<br>Data 2: Skin temperature, blood volume pressure, heart rate, heartbeat inter-beat interval, and electrodermal activity (EDA).	1) Geo-visualization;<br>2) Linear mixed model.	1) DT app allow people to describe aspects of this neighborhood environment that they felt influenced their well-being or the functioning of these public spaces.
13	Dessimond, et al. [13]	L = Paris, France;<br>T = 6.5 or 8 days;	N = 1;<br>A = Adult;	ED = Physical Environment;	Data 1: real-time exposure to air pollutant;	HD = Activity;<br><br>Sensor 2: tablet GPS.	Data 2:<br>GPS trajectories.<br>Activity diary	1) Descriptive Analysis;<br>2) Geo-visualization.	1) online survey

		S = Naturalistic;	G = Not mentioned.	Sensor 1: Canarin.	Contextual: POI data.				
14	Do, et al. [14]	L = Southern California, USA;<br>T = 7 days;<br>S= Naturalistic;	N = 18;<br>A = Adult;<br>G = Both.	ED= Physical Environment;<br><br>Sensor 1: PM monitor;<br>Sensor 2: Temperature logger.	Data 1: Air pollutant PM2.5, PM10 concentration;<br>Data 2: relative humidity (RH) and temperature.<br><br>Contextual: Co-locate the personal monitors at the air monitoring site	HD = Activity;<br><br>Sensor 3: GPS data loggers.<br>Extra: Wi-Fi hotpot.	Data 3: Geo-coordinates.	1) Descriptive Analysis;	
15	Doherty and Oh [15]	L= Toronto, Canada;<br>T = 72 h;<br>S = Naturalistic;	N = 40(37);<br>A = 32/75;<br>G = Both.	ED = urban Environment;<br><br>No particular sensor.	Qualitative data: Subjective assessment from recall diary;	HD= Activity and physical health;<br><br>Sensor 1: GPS receiver;<br>Sensor 2: Electrocardiogram (ECG);<br>Sensor 3: Glucose monitoring;<br><br>A smartphone with custom software.	Data 1: Geo-coordinates;<br>Data 2: Heart rate; Accelerometer;<br>Data 3: Blood glucose.<br><br>Activity data: Subjective data from recall diary.	1) Descriptive statistics;<br>2) Geo-visualization.	1) A food/medicine diary and prompted recall activity diary;<br>2) Prompted recall activity diary, a Web-based interactive interface;<br>3) 2-h interview;<br>4) A follow-up survey asking subjects about their experience.

16	Donaire-Gonzalez, et al. [16]	L = five cities (Amsterdam and Utrecht- the Netherlands, Basel- Switzerland, Norwich-UK, and Torino- Italy); Y = Three times for 24h in three seasons over one year S = Naturalistic;	N = 158; A = Average 61 years old G = Both.	ED = Physical environment; Sensor 1: MicroAeth; Sensor 2 DiSCmini.	Data 1: Black carbon; Data 2: UFP number concentration. Contextual data: Green spaces from Open Street Map;	HD = Activity; Sensor 3 Application ¨ ExpoApp¨; Sensor 4 GPS-Tracker; Sensor 5 Accelerometer.	Data 3: Geo-coordinates and accelerometer; Data 4 and Data 5 for testing. Activity data: Completed a TAD (travel and activity diary | 1) Descriptive statistics.	1) Expo App can integrate and post-process data from portable sensors.
17	Doryab, et al. [17]	L = Los Angeles, USA; T = 16 weeks(one semester); S = Naturalistic;	N = 188 (160); A = College student; G = Both.	ED = Social environment; Sensor 1: Smartphone APP(AWARE).	Data 1: Nearby Bluetooth addresses, Wi-Fi, location, GPS, phone usage (when the screen status changed to on or off and locked or unlocked), and call and short message service (SMS) text messaging logs.	HD = Activity; Sensor 2: A Fitbit Flex 2.	Data 2: The number of steps taken and sleep status (asleep, awake, restless)	1) Descriptive statistics; 2) Aprior, frequent itemset algorithm for discovering associations among items; 3) Machine Learning Analysis.	1) Web-based questionnaires for an initial assessment of their health and well-being; 2) Post measurements.
18	El Aarbaoui and Chaix [18]	L = Paris, France; T = 7 days; S = Naturalistic;	N = 78(75); A = 34–74; G = Both.	ED= Physical Environment; Sensor 1: Personal Dosimeter.	Data 1: The sound level.	HD = Activity and physical health; Sensor 2: GPS receiver; Sensor 3: Actigraph accelerometer. Sensor 4: BioPatch BHM 3 Sensor;	Data 2: Geo-coordinates; Data 3: Accelerometer; Data 4: The HRV/HR.	1) Descriptive statistics; 2) Linear-mixed models.	1) Phone Mobility survey

19	Engelniederhammer, et al. [19]	L = Hongkong, China; T = Mid-noon; S = Walking route with 4 street paths;	N = 30; A = average 24.77 years old G = Both	ED=Social environment; Sensor 1: Panasonic (NaPiOn) with infrared motion sensor;	Data 1: Human presence in an ambient space of up to 2 m distance.	HD = Mental health and psychology; Sensor 2: GPS antenna; Sensor 3: Wristband: body Monitor.	Data 2: Geo-coordinates; Data 3: Skin conductivity and skin temperature.	1) Descriptive statistics; 2) logit models;	App questionnaire.
20	Huck, et al. [20]	L = Lancashire, UK T = days S = A number of different routes, days and times around the city;	N = 1; A = 28 G = Male	ED = Physical environment; Sensor 1: NO_2 sensor 'MiCS-2710'.	Data 1: Ambient NO2 concentration.	HD = Physical health; Sensor 2: Smartphone-based GPS; Sensor 3: 'Airflow Sensor'.	Data 2: Geo-coordinates; Data 3: The frequency and relative depth of breathing with the timestamps.	1) Geo-visualization.	1)Data integrated in a Smartphone software 'Spatial Logger'.
21	Johnston, et al. [21]	L = Southern California, USA; T = average of 18 h per participant; S = Naturalistic;	N = 18(10); A = Youth (15–17); G = Both.	ED = Physical Environment; Sensor 1: Personal PM2.5 monitor; Sensor 2: Phone based sensors.	Data 1: PM2.5 concentrations Data 2: temperature, and relative humidity	HD = Psychology; Sensor 2: Smartphone-based GPS.	Data 2: Geo-coordinates. Psychology: Photographs, videos to document what they saw.	No particular analysis.	1) Popular Education Workshops to cover a broad overview of air pollution. 2) discussion about the questions or ideas.
22	Kanjo, et al. [22]	L = Nottingham, UK; T= 45 mins S = Shopping route;	N = 40; A = 28; G = Female.	ED = Physical environment; Sensor 1: Smartphone; Sensor 2: Microsoft band.	Data 1: Noise; Data 2: Air pressure Light (UV).	HD = Mental health and Psychology; Sensor 1: Smartphone; Sensor 2: Microsoft band.	Data 1: Geo-coordinates; Data 2: 1) Heart Rate (HR), 2) Electro Dermal Activities (EDA) 3) Body Temperature	1) Principle Component Analysis (PCA); 2)Multi-variant regression analysis;	

							4) Hand Acceleration.<br><br>Self-Report of Emotion (1–5) from an App "EnvBodySens".		
23	Kim, et al. [23]	L = Lincoln, Nebraska, USA;<br>T = -<br>S = 1.26 km walking route;	N = 30;<br>A = Average 24.2 years ago<br>G = both.	ED = Urban environment;<br><br>No particular sensor.	Qualitative data: subjective assessment about walkability.	HD = Physical health and activity;<br><br>Sensor 1: Smartphone-based application;<br>Sensor 2 Commercial IMU (Motion Studio);<br>Sensor 3: Empatica E4.	Data 1: Geo-coordinates;<br>Data 2: Accelerometer;<br>Data 3: 1) Heart rate, 2) Photoplethysmography (PPG);<br>3) Blood Volume Pulse (BVP);	1) calculation of gait stability and acceleration;<br>3) Point biserial correlation coefficient (rpb)	1) additional explanation of the rating followed a walkability checklist.
24	Kou, et al. [24]	L = Chicago, USA;<br>T = a weekday and a weekend day;<br>S = Naturalistic;	N = 46(33);<br>A = 18–65;<br>G = Both.	ED = Physical Environment;<br><br>Sensor 1: portable sound sensor.	Data 1: Noise level	HD = Activity;<br><br>Sensor 2: GPS-equipped mobile phone.	Data 2: Geo-coordinates.<br><br>Activity data: Activity diaries.	1) Descriptive analysis<br>2) Multilevel logistic regression analysis	1) Survey questionnaire that collected his/her demographic information and self-reported health status
25	Laeremans, et al. [25]	L = Antwerp, Belgium; Barcelona, Spain. London, UK;<br>T = 7 days, three times in different seasons;<br>S = : Naturalistic;	N = 122;<br>A = Average 35 years old<br>G = both.	ED = Physical environment;<br><br>Sensor 1: MicroAeth.	Data 1: AP exposure and level of Black Carbon.	HD = Physical health and activity;<br><br>Sensor 2: SenseWear armband.	Data 2: 1) Heat flux, 2) Galvanic skin response, skin temperature<br>3) Accelerometer.	1) Kenward-Roger's approximation;	1) At the end of each measurement week, conducting a battery of non-

									invasive measurements about HRV, retinal vessel diameters, FeNO, and lung function at a research center in each of the three cities
26	Ma, et al. [26]	L = Beijing,China; T = 48 hours (a workday and a weekend day); S = Naturalistic;	N = 117 (97) residents; A = 18–60; G = Both.	ED = Physical Environment; Sensor 1: SLM-25 Sound Level Meters.	Data 1: real-time noise level	HD = Activity; Sensor 2: Smartphone based GPS	Data 2: Geo-coordinates. Activity data: Two-day activity-travel diary	1) Descriptive statistics; 2) Regression modeling; 3) 3D geo-visualization;	1) A questionnaire
27	Ma, et al. [27]	L = Beijing,China; T = 48 hours (a workday and a weekend day); S = Naturalistic;	N = 117(112) residents; A = 18–60; G = Both.	ED = Physical Environment; Sensor 1: air pollutant sensors.	Data 1: Real-time PM2.5 concentration Contextual: Residence-based monitoring station assessment	HD = Activity; Sensor 2: GPS-equipped smartphone	Data 2: Geo-coordinates. Qualitative data: Self-reported mental health activity-travel diaries.	1) Descriptive statistics; 2) 3D geo-visualization;	1) A questionnaire
28	Millar, et al. [28]	L = F261 cycle highway from Tilburg via Loon op Zand to Waalwij, Netherlands; T = a day;	N = 12; A = half aged 18–24, the remaining half were older 55; G = Both.	ED = Urban Environment; Sensor 1: Camera.	Data 1: Recording participants' view; Contextual: Digital surface model (DSM) and a land use map, three geospatial data sets.	HD = Mental health; Sensor 2: Smartphone GPS App; Sensor 3: Empatica E4.	Data 2: Geo-coordinates; Data 3: Heart Rate; Temperature, Skin conductivity.	1) Descriptive statistics; 2) Multilevel models; 3) Geo-visualization.	

		S = 18 km long between urban and rural;							
29	Novak, et al. [29]	L = Ljubljana,Slovenia; T = 7 days; S = Naturalistic;	N = 2; A = Adult; G = -.	ED = Physical Environment; Sensor 1: PM measuring unit.	Data 1: real-time PM2.5, PM10 concentration.	HD = Physical health; Sensor 2: Activity tracker (SAT).	Data 2: Hart rate.	1) Four models to approach the data; 2) Summary statistics.	Compared personal monitors with values obtained government run AQ station.
30	Ojha, et al. [30]	L = Zürich, Switzerland; T =- S = 1.3 km walking route;	N = 30; A = - G = -	ED = urban and physical environment; Sensor 1: A sensor backpack.	Data 1: 1) Sound level 2) The amount of dust (mg/m^3), 3) Temperature (°C), relative humidity (%), and illuminance (lx). Contextual: Field-of-view based on the GPS information and spatial configuration of the neighborhood by photo.	HD= Mental health; Sensor 2: GPS receiver; Sensor3: Wrist-worn sensor Empatica E4.	Data 2: GPS location; Data 3: Biological signal Electrodermal activity (EDA).	1) REP-Tree (a decision tree) to build a predictive model; 2) ten-fold cross-validation to validate; 3) FURIA a fuzzy rule-based classifier to build inferential modeling; 4) Backward feature elimination (BFE) method to abstract feature; 5) Self- organizing map (SOM).	
31	Rabinovitch, et al. [31]	L = Morgridge, USA; T = 4 school days; twice in two nonconsecutive weeks S = : Naturalistic;	N = 30; A = schoolchildren average 10 years old; G = -	ED = Physical Environment; Sensor 1: Aerosol nephelometer; Sensor 2:	Data 1: PM concentrations; Data 2: Ambient temperature.	HD = Physical health; Sensor 3: GPS receiver; Device: Monitor Doser.	Data 3: Geo-coordinates; health data: School-time albuterol use as the total number of activations.	1) Linear mixed models.	1) Samples were batch assayed for uLTE4. 2) Daily surveys included

				Temperature sensor.				questions determining presence or absence of upper respiratory infection.
32	Resch, et al. [32]	L = Salzburg, Austria; Cologne, Germany T= Within a day; S= Naturalistic;	N = 56; A = over 18; G = Both.	ED = Urban Environment; Sensor 1: GoPro camera.	Data 1: First-person video camera.	HD= Mental health and psychology; Sensor 2: Smartphone-based eDiary app. Sensor 3: Empatica 4; Sensor 4: Zephyr Bioharness;	Data 2: Geo-location; Data 3: GSR and ST; Data 4: Cardiological parameters such as ECG, HRV, and others;	1) Visual analytics; 2) Descriptive analysis;	A geo-located (post-hoc paper–pencil) questionnaire.
33	Roe, et al. [33]	L = Virginia, USA; T = Unassisted walking for 15–20 min; S = Naturalistic;	N = 11; A = age 65; G = Both.	ED = Physical Environment; Sensor 1: Noise sensors; Sensor 2: Airbeam sensors.	Data 1: Noise (dB); Data 2: Air quality.	HD = Physical health, mental health and psychology; Sensor 3: Phone app Sensor 4: Smart watch	Data 3: GPS data and accelerometer; Data 4: Hart rate variability; Walking speed. Self –report: Mood and Subjective well-being;	1) Descriptive analysis; 2) Linear mixed effects models.	1) Pre- and Post-walk assessment
34	Runkle, et al. [34]	L = three sites: Starkville, Boone and Raleigh, United States; Y=5 days; S = Naturalistic; D = Longitudinal.	N = 66 (35); A = 16–35; G = both.	ED = Physical Environment; Sensor 1: Thermochron iButton;	Data 1 (n = 66): Direct exposure to ambient temperature.	HD = Physical health; Sensor 2: Garmin smartwatch	Data 2: Geo-coordinates; Heart rate and collect physiologic monitoring data.	1) Descriptive statistics; 2) Quasi-Poisson regression.	1) a heat illness survey, 2) time activity logs to capture changes in work activity;

35	Rybarczyk, et al. [35]	L = Wuppertal, Germany; T = hours; S = Naturalistic; within 1.1 km²;	N = 28; A = 20–70; G = Both.	HD = Urban Environment; Sensor 1: GoPro Hero 3 and 4	Qualitative data: Roadway conditions Contextual: Land-use types, road type, road speed limit and roadway width, tree density, Bicycle facility type.	HD = Physical health and activity; Sensor 2: tablet-based GPS; Sensor 3: Garmin VívoSmart;	Data 2: GPS location Data 3: heart rate, altimeter, accelerometer.	1) Descriptive statistics; 3) Linear regression model and spatial auto-regression.	1) a short survey,
36	Shoval, et al. [36]	L = Jerusalem, Israel; T = A day; S = Naturalistic settings;	N = 144 (68); A = over 18 G= both.	ED = Urban environment; No particular sensor.	Qualitative data: location-triggered surveys.	HD = Mental health And psychology; Sensor 1: Smartphone GPS; Sensor 2: Wrist-worn sensor Empatica E4; Smartphone application, 'Sensometer'.	Data1: Geo-coordinates; Data 2(n = 68) Skin conductance, heart rate measures, blood pressure, and skin temperature; Emotion data: time-triggered surveys.	1) Geo-visualization; 2) Descriptive statistics.	
37	Steinle, et al. [37]	L = Edinburgh, Scotland; T = Days in winter and summer; S = Naturalistic settings;	N = 17; A = - G = -	ED = Physical Environments; Sensor 1: Dylos DC 1700;	Data1: Particle number counts (PNC). Contextual: Time–activity diaries (TADs) and questions about building and neighbor characteristics.	HD = Activity; Sensor 2: GPS receiver	Data 2: Geo-coordinates. Activity data: Web-based Time–activity diaries (TADs);	1) visualization of data. 2) Descriptive statistics.	1) Follow-up meeting, a web-form questionnaire;

38	West, et al. [38]	L = Nairobi, Kenya; T = two weeks; S = Naturalistic; D = Longitudinal.	N = 6; A = 18–55; G = Both.	HD = Physical Environment; Sensor 1: Dylos 1700.	Data 1: PM concentrations. Contextual: Assessment from Mobile Air Pollution Laboratory.	HD = Psychology; Sensor 2: GPS tracker;	Data 2: GPS trajectories. Perception data: questionnaire.	1) Descriptive statistics; 2) visualization of data.	1) workshops to discuss citizen science and potential actions. 2) Pre- and Post-questionnaire
9	Zhang, *et al.* [39]	L = Guangzhou, China; T = a weekday and a weekend day; S = Naturalistic;	N = 156(138); A = over 18; G = Both.	ED = Physical and social Environment; Sensor 1: Noise sensors; Sensor 2: Air sensors; Sensor 3: Mobile signal detection.	Data 1: SLM-25 Sound Level Meters; Data 2: Real-time PM2.5, temperature and humidity; Data3: Number of mobile phones in the surrounding.	HD = Psychology; Sensor 4: GPS-equipped mobile phones.	Data 4: GPS trajectories. EMA data: Geographic eco-logical momentary assessment (GEMA) of environmental perceptions and emotions.	1) Descriptive statistics; 2) Hierarchical logistic logistic models (HLMs)	1) Daily activity and environmental health questionnaire survey;

Table S2. Checklist for the methodological quality assessment.

Criteria [1]	Description	Score
Study purpose	**Was the purpose and/or research question stated clearly?**	
	No	0
	Yes	1
Literature	**Was relevant background literature reviewed?**	
	No	0
	Yes	1
Sampling	**Was the process of purposeful selection described?**	
	No	0
	Yes (e.g., probability sampling methods were used)	1
	Are **the individuals selected to participate in the study likely to be representative of the target population?/was sample size justified?**	
	Weak	0
	Moderate	1
	Strong	2
	**Was informed consent obtained?**	
	No	0
	Yes	1
Study Design [2]	Cross-sectional study 1 (Minutes/ Hours within a day)	0
	Cross-sectional study 2 (Days/Weeks)	1
	Longitudinal study (Months and Seasons)	2
Data Collection Method	**Clear** and **complete description about site and participants**	
	No	0
	Yes	1
	**Was the measurement tool used valid and reliable (humans' responses)?**	
	Weak	0
	Moderate	1
	Strong	2
	**Was the measurement tool used valid and reliable (environment independent variable)?**	
	Weak	0
	Moderate	1
	Strong	2
Withdrawals	**Were drop-outs/exclusion reported in terms of numbers and/or reasons per group?**	
	No	0
	Yes	1
Confounders	**Were relevant confounders controlled?**	
	Weak	0
	Moderate	1
	Strong	2
Data Analysis	**Were the statistical methods appropriate for the study design?**	
	Weak	0
	Moderate	1
	Strong	2
	**Was the process of analysing data described adequately?**	
	No	0
	Yes	1
	**Were conclusions appropriate given the study findings?**	

Conclusion and Implications	No	0
	Yes	1

[1] The checklist was adapted from previously established assessment tools (EPHPP,2010; Law al.,1998), this adaption was used by Won et al (2016). [2] This review focuses on the measurement of environment and health in the fieldwork, rather than medical research in lab, so we use the criteria of Study design adapted by Won et al (2016).

Table S3. Quality assessment of the reviewed studies. MT-Human: measurement tool used for human (0-No; 1- Traditional & subjective data; 2- Objective measurement such as GPS, accelerometer, health tracker). DD-Human: Data dimension of objectively measuring humans' responses (0- Low-dimensions; 1- High- dimensions). MT- Environment: measurement tool used for Environment (0-No; 1- Traditional & subjective data; 2- Objective measurement such as personalized environmental sensors). DD- Environment: Data dimension of objectively measuring environment (0- Low-dimensions; 1- High- dimensions).

No.	RE	Purpose	Literature	Sampling			Study Design	Data Collection			Withdrawals	Confounders	Data Analysis		Conclusion	Total Score
				Purpose	Representation	Consent		Description	Health	Environment			Method	Process		
1	Benita, et al. [1]	1	1	1	0	1	0	1	2	2	0	2	2	1	1	15(M)
2	Benita and Tunçer [2]	1	1	1	0	1	0	1	2	2	0	2	2	1	1	15(M)
3	Birenboim, et al. [3]	1	1	0	0	1	0	1	2	1	1	2	0	1	1	12(M)
4	Bohmer, et al. [4]	1	1	1	2	1	1	1	2	2	1	1	0	1	1	16(H)
5	Boissy, et al. [5]	1	1	1	2	1	1	1	2	1	1	1	1	1	1	16(H)
6	Bolliger, et al. [6]	1	1	1	0	1	1	1	2	2	0	1	0	0	0	11(L)
7	Borghi, et al. [7]	1	1	0	0	0	2	1	2	2	0	0	0	1	0	10(L)
8	Burgi, et al. [8]	1	1	1	2	1	1	1	2	1	1	1	0	1	1	15(M)
9	Butt, et al. [9]	1	1	1	0	1	1	1	2	2	1	0	2	1	1	15(M)
10	Cerin, et al. [10]	1	1	1	2	1	1	1	2	1	1	2	1	1	1	17(H)
11	Chaix, et al. [11]	1	1	1	2	1	1	1	2	1	1	1	0	1	1	15(M)
12	Chrisinger and King [12]	1	1	1	0	1	0	1	2	1	0	1	1	1	1	12(M)
13	Dessimond, et al. [13]	1	1	0	0	1	1	1	1	2	0	0	0	1	1	10(L)
14	Do, et al. [14]	1	1	0	0	0	1	1	0	2	0	1	0	1	1	9(L)
15	Doherty and Oh [15]	1	1	1	1	0	1	1	2	1	1	0	0	1	1	12(M)
16	Donaire-Gonzalez, et al. [16]	1	1	0	2	1	2	1	2	2	0	1	0	1	1	15(M)
17	Doryab, et al. [17]	1	1	1	2	1	2	1	2	2	1	1	2	1	1	19(H)
18	El Aarbaoui and Chaix [18]	1	1	1	2	1	1	1	2	2	1	1	1	1	1	17(H)
19	Engelniederhammer, et al. [19]	1	1	1	1	0	0	1	2	2	0	1	1	1	1	13(M)
20	Huck, et al. [20]	1	1	0	0	0	1	1	2	2	0	0	0	1	1	10(L)
21	Johnston, et al. [21]	1	0	0	0	0	1	1	1	2	1	0	0	0	1	8(L)
22	Kanjo, et al. [22]	1	1	1	1	1	0	1	2	2	0	2	2	1	1	16(H)
23	Kim, et al. [23]	1	1	0	1	0	0	1	2	1	0	2	1	1	1	12(M)

24	Kou, et al. [24]	1	1	0	1	1	1	1	2	1	1	2	1	1	1	15(M)
25	Laeremans, et al. [25]	1	1	1	2	1	2	1	2	2	0	2	1	1	1	18(H)
26	Ma, et al. [26]	1	1	0	2	1	1	1	1	2	1	2	1	1	1	16(H)
27	Ma, et al. [27]	1	1	0	2	0	1	1	1	2	1	1	0	1	1	13(M)
28	Millar, et al. [28]	1	1	1	0	0	0	1	2	2	0	1	1	1	1	12(M)
29	Novak, et al. [29]	1	1	0	0	0	1	1	2	2	0	0	2	1	1	12(M)
30	Ojha, et al. [30]	1	1	0	1	0	0	1	2	2	0	1	2	1	1	13(M)
31	Rabinovitch, et al. [31]	1	0	1	1	1	1	1	2	2	0	1	1	1	1	14(M)
32	Resch, et al. [32]	1	1	0	1	1	0	1	2	2	0	1	1	1	1	13(M)
33	Roe, et al. [33]	1	1	1	0	1	0	1	2	2	0	2	1	1	1	14(M)
34	Runkle, et al. [34]	1	1	1	1	1	1	1	2	2	1	2	1	1	1	17(H)
35	Rybarczyk, et al. [35]	1	1	0	1	1	0	1	2	2	0	1	2	1	1	14(M)
36	Shoval, et al. [36]	1	1	1	2	1	0	1	2	1	1	1	0	1	1	14(M)
37	Steinle, et al. [37]	1	1	0	0	0	2	1	1	2	0	1	0	1	1	11(L)
38	West, et al. [38]	1	1	1	0	1	1	1	1	2	0	0	0	1	1	11(L)
39	Zhang, et al. [39]	1	1	1	2	1	1	1	1	2	1	1	1	1	1	16(H)

Table S4. Sensors and devices in the reviewed studies.

Topic	Sensor		Index	Reference
Health tracker to monitor human responses	Wristband	Empatica (E4) [1]	Body skin temperature and Electrodermal activity (EDA), etc.	Benita, et al. [1] Benita and Tunçer [2] Bolliger, et al. [6] Chrisinger and King [12] Kim, et al. [23] Millar, et al. [28] Ojha, et al. [30] Resch, et al. [32] Shoval, et al. [36].
		Microsoft Band [2] (MS Band)	Heart rate (HR), Electrodermal activity (EDA), skin temperature, blood volume pulse (BVP), etc.	Birenboim, et al. [3] Kanjo, et al. [22].
		A Fitbit Flex [3] (Fitbit)	The number of steps taken and sleep status (asleep, awake, restless), etc.	Doryab, et al. [17]
		Garmin [4]	Heart rate (HR), steps, etc.	Novak, et al. [29] Runkle, et al. [34] Rybarczyk, et al. [35].
		Heart rate monitor	Heart beat (HB), etc.	Borghi, et al. [7] Rybarczyk, et al. [35].

		(Suunto 9 [5])		
		Body monitor wristband	Skin conductivity and skin temperature, etc.	Engelniederhammer, et al. [19].
		Huawei watch	Photoplethysmogram (PPG)	Roe, et al. [33].
	Armband	SenseWear [6]	Heat flux, galvanic skin response, skin temperature, etc.	Laeremans, et al. [25].
	Chest Strap	BioPatch BHM 3	Electrocardiogram (ECG) signal	El Aarbaoui and Chaix [18].
		Zephyr Bioharness [7]	Electrocardiogram (ECG) signal	Resch, et al. [32].
	Other medical sensors	Alive Heart Monitor [8]	Electrocardiogram (ECG) signal	Doherty and Oh [15].
		Glucose monitor (Medtronic [9])	Blood glucose, etc.	Doherty and Oh [15].
		Airflow Sensor	The frequency and relative depth of breathing, etc.	Huck, et al. [20].
		Sleep monitor (ZEO [10])	Total sleep time (TST), rapid eye movement (REM), etc.	Butt, et al. [9].
Motion	physical activity	Accelerometer	Acceleration, speed and direction, etc.	Bohmer, et al. [4] Burgi, et al. [8] Cerin, et al. [10] Chaix, et al. [11] Doherty and Oh [15] Donaire-Gonzalez, et al. [16] El Aarbaoui and Chaix [18] Kim, et al. [23] Laeremans, et al. [25].
Environment sensors to measure exposure		Wind sensor	Wind speed.	Benita and Tunçer [2]
		Air Pressure sensor	Atmospheric pressure, etc.	Benita, et al. [1] Benita and Tunçer [2] Kanjo, et al. [22]
		Light sensor	Illuminance, Light exposure etc.	Bohmer, et al. [4] Kanjo, et al. [22] Ojha, et al. [30]
		Sound sensor	Noise level, etc.	Benita, et al. [1] Benita and Tunçer [2] El Aarbaoui and Chaix [18] Johnston, et al. [21] Kou, et al. [24] Ma, et al. [26] Ojha, et al. [30] Roe, et al. [33] Zhang, et al. [39]
		Temperature sensor	Air temperature, relative humidity, etc.	Benita, et al. [1] Benita and Tunçer [2] Do, et al. [14] Ojha, et al. [30] Rabinovitch, et al. [31] Runkle, et al. [34]
		Dust sensor	The level of dust in the air	Ojha, et al. [30]
		Infrared motion sensor	Distance and Density, etc.	Engelniederhammer, et al. [19]
		camera	View, etc.	Millar, et al. [28] Resch, et al. [32] Rybarczyk, et al. [35].

	Nitrogen Dioxide (NO^2) sensor	Ambient NO2 concentration, etc.	Borghi, et al. [7] Huck, et al. [20] Kanjo, et al. [22]
	Particulate Matter (PM)	The concentration of particulars number , such as PM1, PM2.5, PM10, etc.	Dessimond, et al. [13] Do, et al. [14] Donaire-Gonzalez, et al. [16] Johnston, et al. [21] Laeremans, et al. [25] Ma, et al. [27]<br>Novak, et al. [29] Ojha, et al. [30] Rabinovitch, et al. [31] Roe, et al. [33] Steinle, et al. [37] West, et al. [38] Zhang, et al. [39]
	Ultrafine Particles (UFPs]	UFP exposure levels	Borghi, et al. [7] Donaire-Gonzalez, et al. [16]
	Black carbon monitor	Black carbon levels	Donaire-Gonzalez, et al. [16] Laeremans, et al. [25]
GPS	GPS receiver	Geographic coordinates.	Birenboim, et al. [3] Boissy, et al. [5] Burgi, et al. [8] Cerin, et al. [10] Chaix, et al. [11] Do, et al. [14] Doherty and Oh [15] Donaire-Gonzalez, et al. [16] El Aarbaoui and Chaix [18] Engelniederhammer, et al. [19] Huck, et al. [20] Ojha, et al. [30] Rabinovitch, et al. [31] Runkle, et al. [34] Steinle, et al. [37] West, et al. [38]
	Sensor-inbuilt GPS	Geographic coordinates.	Borghi, et al. [7]
Smartphone based	Geo-coordinates	Position service.	Benita, et al. [1] Benita and Tunçer [2] Bolliger, et al. [6] Chaix, et al. [11] Chrisinger and King [12] Dessimond, et al. [13] Donaire-Gonzalez, et al. [16] Bolliger, et al. [6] Huck, et al. [20] Johnston, et al. [21] Kanjo, et al. [22] Kim, et al. [23] Kou, et al. [24] Ma, et al. [26] Ma, et al. [27] Millar, et al. [28] Resch, et al. [32] Roe, et al. [33] Shoval, et al. [36] Rybarczyk, et al. [35] Zhang, et al. [39]
	Accelerometer	Acceleration, speed and direction, etc.	Donaire-Gonzalez, et al. [16] Roe, et al. [33]
	Wifi	Location; network	Do, et al. [14]
	Light	Luminance of the ambient light	Bolliger, et al. [6]
	Microphone	Voice activity; noise	Benita, et al. [1] Benita and Tunçer [2] Bolliger, et al. [6] Kanjo, et al. [22]
	Bluetooth	Proximity; Social communication	Bolliger, et al. [6] Doryab, et al. [17]
	Images	Narratives and views.	Chrisinger and King [12]
	Temperature	Temperature of the phone's hardware sensor	Bolliger, et al. [6] Johnston, et al. [21]

Application	Such as ¨FunF sensing platform¨, ¨ExpoApp¨, "eDiary", ¨Aware¨, ¨Sensometer', etc.	Butt, et al. [9] Chrisinger and King [12] Donaire-Gonzalez, et al. [16] Doryab, et al. [17] Kim, et al. [23] Shoval, et al. [36] Resch, et al. [32]

[1] Empatica, more information: https://www.empatica.com/en-int/ (accessed on 8 November 2021); [2] Microsoft Band, more information: https://en.wikipedia.org/wiki/Microsoft_Band. [3] Fibit, more information: https://www.fitbit.com/global/dk/home (accessed on 8 November 2021); [4] Garmin, more information: https://www.garmin.com/en-US/ (accessed on 8 November 2021); [5] Suunto 9, more information: https://www.suunto.com/da-dk/suunto-collections/suunto-9-collection/ (accessed on 8 November 2021). [6] SenseWear, more information: chrome-e√tension://ohfgljdgelakfkefopgklcohadegdpjf/http://nutrizionista-gorizia.it/resources/MicroStar---Rheo-Scan---D-300.pdf (accessed on 8 November 2021); [7] Zephyr Bioharness, more information: https://www.zephyranywhere.com/ (accessed on 8 November 2021). [8] Alive Heart Monitor, more information: https://www.kardia.com/ (accessed on 8 November 2021). [9] Medtronic, more information: https://www.medtronicdiabetes.com/treatments/continuous-glucose-monitoring (accessed on 8 November 2021); [10] Zeo sleep tracking, more information: https://polyphasic.net/sleep-tracking/zeo-sleep-tracking/ (accessed on 8 November 2021).

References

1. Lazarus, R.S.; Cohen, J.B. Environmental Stress. In *Human Behavior and Environment: Advances in Theory and Research*; Altman, I., Wohlwill, J.F., Eds.; Springer: Boston, MA, USA,1977; Volume 2; pp. 89–127.
2. Ali, M.U.; Liu, G.; Yousaf, B.; Ullah, H.; Abbas, Q.; Munir, M.A.M. A systematic review on global pollution status of particulate matter-associated potential toxic elements and health perspectives in urban environment. *Environ. Geochem. Health* **2019**, *41*, 1131–1162, doi:10.1007/s10653-018-0203-z.
3. Sygna, K.; Aasvang, G.M.; Aamodt, G.; Oftedal, B.; Krog, N.H. Road traffic noise, sleep and mental health. *Environ. Res.* **2014**, *131*, 17–24, doi:10.1016/j.envres.2014.02.010.
4. Lopez, R.P.; Hynes, H.P. Obesity, physical activity, and the urban environment: Public health research needs. *Environ. Health* **2006**, *5*, 25, doi:10.1186/1476-069X-5-25.
5. Swan, M. The Quantified Self: Fundamental Disruption in Big Data Science and Biological Discovery. *Big Data* **2013**, *1*, 85–99, doi:10.1089/big.2012.0002.
6. Tung, J.Y.; Rose, R.V.; Gammada, E.; Lam, I.; Roy, E.A.; Black, S.E.; Poupart, P. Measuring life space in older adults with mild-to-moderate Alzheimer's disease using mobile phone GPS. *Gerontology* **2014**, *60*, 154–162, doi:10.1159/000355669.
7. MacKerron, G.; Mourato, S. Happiness is greater in natural environments. *Glob. Environ. Chang.* **2013**, *23*, 992–1000, doi:10.1016/j.gloenvcha.2013.03.010.
8. Li, X.; Dunn, J.; Salins, D.; Zhou, G.; Zhou, W.; Schussler-Fiorenza Rose, S.M.; Perelman, D.; Colbert, E.; Runge, R.; Rego, S.; et al. Digital Health: Tracking Physiomes and Activity Using Wearable Biosensors Reveals Useful Health-Related Information. *PLoS Biol.* **2017**, *15*, e2001402, doi:10.1371/journal.pbio.2001402.
9. Ermes, M.; Parkka, J.; Mantyjarvi, J.; Korhonen, I. Detection of Daily Activities and Sports With Wearable Sensors in Controlled and Uncontrolled Conditions. *IEEE Trans. Inf. Technol. Biomed.* **2008**, *12*, 20–26, doi:10.1109/titb.2007.899496.
10. Gorgul, E.; Zhang, L.; Günther, F.; Chen, C. Mapping Human Response to Street Experience. In Proceedings of Adjunct Proceedings of the 2019 ACM International Joint Conference on Pervasive and Ubiquitous Computing and Proceedings of the 2019 ACM International Symposium on Wearable Computers, London, UK, 9–13 September 2019; pp. 69–72.
11. Morawska, L.; Thai, P.K.; Liu, X.; Asumadu-Sakyi, A.; Ayoko, G.; Bartonova, A.; Bedini, A.; Chai, F.; Christensen, B.; Dunbabin, M.; et al. Applications of low-cost sensing technologies for air quality monitoring and exposure assessment: How far have they gone? *Environ. Int.* **2018**, *116*, 286–299, doi:10.1016/j.envint.2018.04.018.
12. Vlachokostas, C.; Achillas, C.; Michailidou, A.V.; Moussiopoulos, N. Measuring combined exposure to environmental pressures in urban areas: An air quality and noise pollution assessment approach. *Environ. Int.* **2012**, *39*, 8–18, doi:10.1016/j.envint.2011.09.007.
13. Hedendahl, L.K.; Carlberg, M.; Koppel, T.; Hardell, L. Measurements of Radiofrequency Radiation with a Body-Borne Exposimeter in Swedish Schools with Wi-Fi. *Front. Public Health* **2017**, *5*, 279, doi:10.3389/fpubh.2017.00279.
14. Benita, F.; Bansal, G.; Tuncer, B. Public spaces and happiness: Evidence from a large-scale field experiment. *Health Place* **2019**, *56*, 9–18, doi:10.1016/j.healthplace.2019.01.014.
15. Mamun, M.A.A.; Yuce, M.R. Sensors and Systems for Wearable Environmental Monitoring Toward IoT-Enabled Applications: A Review. *IEEE Sens. J.* **2019**, *19*, 7771–7788, doi:10.1109/jsen.2019.2919352.
16. Riazul Islam, S.M.; Daehan, K.; Humaun Kabir, M.; Hossain, M.; Kyung-Sup, K. The Internet of Things for Health Care: A Comprehensive Survey. *IEEE Access* **2015**, *3*, 678–708, doi:10.1109/access.2015.2437951.
17. Windmiller, J.R.; Wang, J. Wearable Electrochemical Sensors and Biosensors: A Review. *Electroanalysis* **2013**, *25*, 29–46, doi:10.1002/elan.201200349.
18. Qi, J.; Yang, P.; Min, G.; Amft, O.; Dong, F.; Xu, L. Advanced internet of things for personalised healthcare systems: A survey. *Pervasive Mobile Comput.* **2017**, *41*, 132–149, doi:10.1016/j.pmcj.2017.06.018.
19. EPHPP. Quality assessment tool for quantitative studies. Effective Public Health Practice Project. 2010. Available online http://www.ephpp.ca/index.html%5Cnhttp://www.ephpp.ca/PDF/Quality Assessment Tool_2010_2.pdf (accessed on 8 November^{h} 2021)
20. Law, M.; Stewart, D.; Letts, L.; Pollock, N.; Bosch, J.; Westmorland, M. Guidelines for Critical Review of Qualitative Studies. 1998. Available online https://tbzmed.ac.ir/Uploads/3/cms/user/File/10/Pezeshki_Ejtemaei/conferance/dav.pdf (accessed on 8 November 2021)
21. Armijo-Olivo, S.; Stiles, C.R.; Hagen, N.A.; Biondo, P.D.; Cummings, G.G. Assessment of study quality for systematic reviews: A comparison of the Cochrane Collaboration Risk of Bias Tool and the Effective Public Health Practice Project Quality Assessment Tool: Methodological research. *J. Eval. Clin. Pract.* **2012**, *18*, 12–18, doi:10.1111/j.1365-2753.2010.01516.x.
22. Driessen, C.E.; Cameron, A.J.; Thornton, L.E.; Lai, S.K.; Barnett, L.M. Effect of changes to the school food environment on eating behaviours and/or body weight in children: A systematic review. *Obes. Rev.* **2014**, *15*, 968–982, doi:10.1111/obr.12224.
23. van den Bogerd, N.; Coosje Dijkstra, S.; Koole, S.L.; Seidell, J.C.; de Vries, R.; Maas, J. Nature in the indoor and outdoor study environment and secondary and tertiary education students' well-being, academic outcomes, and possible mediating pathways: A systematic review with recommendations for science and practice. *Health Place* **2020**, *66*, 102403, doi:10.1016/j.healthplace.2020.102403.

24. Smith, M.; Hosking, J.; Woodward, A.; Witten, K.; MacMillan, A.; Field, A.; Baas, P.; Mackie, H. Systematic literature review of built environment effects on physical activity and active transport—An update and new findings on health equity. *Int. J. Behav. Nutr. Phys. Act.* **2017**, *14*, 158, doi:10.1186/s12966-017-0613-9.
25. Wang, L.; Wen, C. The Relationship between the Neighborhood Built Environment and Active Transportation among Adults: A Systematic Literature Review. *Urban Sci.* **2017**, *1*, 29, doi:10.3390/urbansci1030029.
26. Won, J.; Lee, C.; Forjuoh, S.N.; Ory, M.G. Neighborhood safety factors associated with older adults' health-related outcomes: A systematic literature review. *Soc. Sci. Med.* **2016**, *165*, 177–186, doi:10.1016/j.socscimed.2016.07.024.
27. Benita, F.; Bansal, G.; Virupaksha, D.; Scandola, F.; Tuncer, B. Body responses towards a morning walk in a tropical city. *Landsc. Res.* **2020**, *45*, 966–983, doi:10.1080/01426397.2020.1808956.
28. Benita, F.; Tunçer, B. Exploring the effect of urban features and immediate environment on body responses. *Urban For. Urban Green.* **2019**, *43*, doi:10.1016/j.ufug.2019.126365.
29. Birenboim, A.; Dijst, M.; Scheepers, F.E.; Poelman, M.P.; Helbich, M. Wearables and Location Tracking Technologies for Mental-State Sensing in Outdoor Environments. *Prof. Geogr.* **2019**, *71*, 449–461, doi:10.1080/00330124.2018.1547978.
30. Bohmer, M.N.; Valstar, M.J.; Aarts, M.P.J.; Bindels, P.J.E.; Oppewal, A.; van Someren, E.J.W.; Festen, D.A.M. Shedding light on light exposure in elderly with intellectual disabilities. *J. Intellect. Disabil. Res.* **2021**, *65*, 361–372, doi:10.1111/jir.12822.
31. Boissy, P.; Blamoutier, M.; Briere, S.; Duval, C. Quantification of Free-Living Community Mobility in Healthy Older Adults Using Wearable Sensors. *Front. Public Health* **2018**, *6*, 216, doi:10.3389/fpubh.2018.00216.
32. Bolliger, L.; Lukan, J.; Lustrek, M.; De Bacquer, D.; Clays, E. Protocol of the STRess at Work (STRAW) Project: How to Disentangle Day-to-Day Occupational Stress among Academics Based on EMA, Physiological Data, and Smartphone Sensor and Usage Data. *Int. J. Environ. Res. Public Health* **2020**, *17*, 8835, doi:10.3390/ijerph17238835.
33. Borghi, F.; Spinazze, A.; Fanti, G.; Campagnolo, D.; Rovelli, S.; Keller, M.; Cattaneo, A.; Cavallo, D.M. Commuters' Personal Exposure Assessment and Evaluation of Inhaled Dose to Different Atmospheric Pollutants. *Int. J. Environ. Res. Public Health* **2020**, *17*, 3357, doi:10.3390/ijerph17103357.
34. Burgi, R.; Tomatis, L.; Murer, K.; de Bruin, E.D. Localization of Physical Activity in Primary School Children Using Accelerometry and Global Positioning System. *PLoS ONE* **2015**, *10*, e0142223, doi:10.1371/journal.pone.0142223.
35. Butt, M.; Ouarda, T.B.; Quan, S.F.; Pentland, A.S.; Khayal, I. Technologically sensed social exposure related to slow-wave sleep in healthy adults. *Sleep Breath* **2015**, *19*, 255–261, doi:10.1007/s11325-014-1005-x.
36. Cerin, E.; Baranowski, T.; Barnett, A.; Butte, N.; Hughes, S.; Lee, R.E.; Mendoza, J.A.; Thompson, D.; O'Connor, T.M. Places where preschoolers are (in)active: An observational study on Latino preschoolers and their parents using objective measures. *Int. J. Behav. Nutr. Phys. Act.* **2016**, *13*, 29, doi:10.1186/s12966-016-0355-0.
37. Chaix, B.; Benmarhnia, T.; Kestens, Y.; Brondeel, R.; Perchoux, C.; Gerber, P.; Duncan, D.T. Combining sensor tracking with a GPS-based mobility survey to better measure physical activity in trips: Public transport generates walking. *Int. J. Behav. Nutr. Phys. Act.* **2019**, *16*, 84, doi:10.1186/s12966-019-0841-2.
38. Chrisinger, B.W.; King, A.C. Stress experiences in neighborhood and social environments (SENSE): A pilot study to integrate the quantified self with citizen science to improve the built environment and health. *Int. J. Health Geogr.* **2018**, *17*, 17, doi:10.1186/s12942-018-0140-1.
39. Dessimond, B.; Annesi-Maesano, I.; Pepin, J.L.; Srairi, S.; Pau, G. Academically Produced Air Pollution Sensors for Personal Exposure Assessment: The Canarin Project. *Sensors* **2021**, *21*, 1876, doi:10.3390/s21051876.
40. Do, K.; Yu, H.; Velasquez, J.; Grell-Brisk, M.; Smith, H.; Ivey, C.E. A data-driven approach for characterizing community scale air pollution exposure disparities in inland Southern California. *J. Aerosol Sci.* **2021**, *152*, doi:10.1016/j.jaerosci.2020.105704.
41. Doherty, S.T.; Oh, P. A multi-sensor monitoring system of human physiology and daily activities. *Telemed. J. E Health* **2012**, *18*, 185–192, doi:10.1089/tmj.2011.0138.
42. Donaire-Gonzalez, D.; Valentin, A.; van Nunen, E.; Curto, A.; Rodriguez, A.; Fernandez-Nieto, M.; Naccarati, A.; Tarallo, S.; Tsai, M.Y.; Probst-Hensch, N.; et al. ExpoApp: An integrated system to assess multiple personal environmental exposures. *Environ. Int.* **2019**, *126*, 494–503, doi:10.1016/j.envint.2019.02.054.
43. Doryab, A.; Villalba, D.K.; Chikersal, P.; Dutcher, J.M.; Tumminia, M.; Liu, X.; Cohen, S.; Creswell, K.; Mankoff, J.; Creswell, J.D.; et al. Identifying Behavioral Phenotypes of Loneliness and Social Isolation with Passive Sensing: Statistical Analysis, Data Mining and Machine Learning of Smartphone and Fitbit Data. *JMIR Mhealth Uhealth* **2019**, *7*, e13209, doi:10.2196/13209.
44. El Aarbaoui, T.; Chaix, B. The short-term association between exposure to noise and heart rate variability in daily locations and mobility contexts. *J. Exp. Sci. Environ. Epidemiol.* **2020**, *30*, 383–393, doi:10.1038/s41370-019-0158-x.
45. Engelniederhammer, A.; Papastefanou, G.; Xiang, L. Crowding density in urban environment and its effects on emotional responding of pedestrians: Using wearable device technology with sensors capturing proximity and psychophysiological emotion responses while walking in the street. *J. Hum. Behav. Soc. Environ.* **2019**, *29*, 630–646, doi:10.1080/10911359.2019.1579149.
46. Huck, J.J.; Whyatt, J.D.; Coulton, P.; Davison, B.; Gradinar, A. Combining physiological, environmental and locational sensors for citizen-oriented health applications. *Environ. Monit. Assess.* **2017**, *189*, 114, doi:10.1007/s10661-017-5817-6.
47. Johnston, J.E.; Juarez, Z.; Navarro, S.; Hernandez, A.; Gutschow, W. Youth Engaged Participatory Air Monitoring: A 'Day in the Life' in Urban Environmental Justice Communities. *Int. J. Environ. Res. Public Health* **2019**, *17*, 93, doi:10.3390/ijerph17010093.
48. Kanjo, E.; Younis, E.M.G.; Sherkat, N. Towards unravelling the relationship between on-body, environmental and emotion data using sensor information fusion approach. *Inf. Fusion* **2018**, *40*, 18–31, doi:10.1016/j.inffus.2017.05.005.

49. Kim, J.; Ahn, C.R.; Nam, Y. The influence of built environment features on crowdsourced physiological responses of pedestrians in neighborhoods. *Comput. Environ. Urban Syst.* **2019**, *75*, 161–169, doi:10.1016/j.compenvurbsys.2019.02.003.
50. Kou, L.R.; Kwan, M.P.; Chai, Y.W. The effects of activity-related contexts on individual sound exposures: A time-geographic approach to soundscape studies. *Environ. Plan. B-Urban Anal. City Sci.* **2020**, *48*, doi:10.1177/2399808320965243.
51. Laeremans, M.; Dons, E.; Avila-Palencia, I.; Carrasco-Turigas, G.; Orjuela, J.P.; Anaya, E.; Cole-Hunter, T.; de Nazelle, A.; Nieuwenhuijsen, M.; Standaert, A.; et al. Short-term effects of physical activity, air pollution and their interaction on the cardiovascular and respiratory system. *Environ. Int.* **2018**, *117*, 82–90, doi:10.1016/j.envint.2018.04.040.
52. Ma, J.; Li, C.J.; Kwan, M.P.; Kou, L.R.; Chai, Y.W. Assessing personal noise exposure and its relationship with mental health in Beijing based on individuals' space-time behavior. *Environ. Int.* **2020**, *139*, doi:10.1016/j.envint.2020.105737.
53. Ma, J.; Tao, Y.; Kwan, M.-P.; Chai, Y. Assessing Mobility-Based Real-Time Air Pollution Exposure in Space and Time Using Smart Sensors and GPS Trajectories in Beijing. *Ann. Am. Assoc. Geogr.* **2019**, *110*, 434–448, doi:10.1080/24694452.2019.1653752.
54. Millar, G.C.; Mitas, O.; Boode, W.; Hoeke, L.; de Kruijf, J.; Petrasova, A.; Mitasova, H. Space-time analytics of human physiology for urban planning. *Comput. Environ. Urban Syst.* **2021**, *85*, 101554, doi:10.1016/j.compenvurbsys.2020.101554.
55. Novak, R.; Kocman, D.; Robinson, J.A.; Kanduc, T.; Sarigiannis, D.; Horvat, M. Comparing Airborne Particulate Matter Intake Dose Assessment Models Using Low-Cost Portable Sensor Data. *Sensors* **2020**, *20*, 1406, doi:10.3390/s20051406.
56. Ojha, V.K.; Griego, D.; Kuliga, S.; Bielik, M.; Buš, P.; Schaeben, C.; Treyer, L.; Standfest, M.; Schneider, S.; König, R.; et al. Machine learning approaches to understand the influence of urban environments on human's physiological response. *Inf. Sci.* **2019**, *474*, 154–169, doi:10.1016/j.ins.2018.09.061.
57. Rabinovitch, N.; Adams, C.D.; Strand, M.; Koehler, K.; Volckens, J. Within-microenvironment exposure to particulate matter and health effects in children with asthma: A pilot study utilizing real-time personal monitoring with GPS interface. *Environ. Health* **2016**, *15*, 96, doi:10.1186/s12940-016-0181-5.
58. Resch, B.; Puetz, I.; Bluemke, M.; Kyriakou, K.; Miksch, J. An Interdisciplinary Mixed-Methods Approach to Analyzing Urban Spaces: The Case of Urban Walkability and Bikeability. *Int. J. Environ. Res. Public Health* **2020**, *17*, 6994, doi:10.3390/ijerph17196994.
59. Roe, J.; Mondschein, A.; Neale, C.; Barnes, L.; Boukhechba, M.; Lopez, S. The Urban Built Environment, Walking and Mental Health Outcomes Among Older Adults: A Pilot Study. *Front. Public Health* **2020**, 8:575946, doi:10.3389/fpubh.2020.575946.
60. Runkle, J.D.; Cui, C.; Fuhrmann, C.; Stevens, S.; Del Pinal, J.; Sugg, M.M. Evaluation of wearable sensors for physiologic monitoring of individually experienced temperatures in outdoor workers in southeastern U.S. *Environ. Int.* **2019**, *129*, 229–238, doi:10.1016/j.envint.2019.05.026.
61. Rybarczyk, G.; Ozbil, A.; Andresen, E.; Hayes, Z. Physiological responses to urban design during bicycling: A naturalistic investigation. *Transp. Res. Part F Traffic Psychol. Behav.* **2020**, *68*, 79–93, doi:10.1016/j.trf.2019.12.001.
62. Shoval, N.; Schvimer, Y.; Tamir, M. Tracking technologies and urban analysis: Adding the emotional dimension. *Cities* **2018**, *72*, 34–42, doi:10.1016/j.cities.2017.08.005.
63. Steinle, S.; Reis, S.; Sabel, C.E.; Semple, S.; Twigg, M.M.; Braban, C.F.; Leeson, S.R.; Heal, M.R.; Harrison, D.; Lin, C.; et al. Personal exposure monitoring of PM2.5 in indoor and outdoor microenvironments. *Sci. Total Environ.* **2015**, *508*, 383–394, doi:10.1016/j.scitotenv.2014.12.003.
64. West, S.E.; Büker, P.; Ashmore, M.; Njoroge, G.; Welden, N.; Muhoza, C.; Osano, P.; Makau, J.; Njoroge, P.; Apondo, W. Particulate matter pollution in an informal settlement in Nairobi: Using citizen science to make the invisible visible. *Appl. Geogr.* **2020**, *114*, doi:10.1016/j.apgeog.2019.102133.
65. Zhang, X.; Zhou, S.H.; Kwan, M.P.; Su, L.L.; Lu, J.W. Geographic Ecological Momentary Assessment (GEMA) of environmental noise annoyance: The influence of activity context and the daily acoustic environment. *Int. J. Health Geogr.* **2020**, *19*, 50, https://doi.org/10.1186/s12942-020-00246-w.
66. St Fleur, R.G.; St George, S.M.; Leite, R.; Kobayashi, M.; Agosto, Y.; Jake-Schoffman, D.E. Use of Fitbit Devices in Physical Activity Intervention Studies Across the Life Course: Narrative Review. *JMIR Mhealth Uhealth* **2021**, *9*, e23411, doi:10.2196/23411.
67. Vich, G.; Marquet, O.; Miralles-Guasch, C. Green exposure of walking routes and residential areas using smartphone tracking data and GIS in a Mediterranean city. *Urban For. Urban Green.* **2019**, *40*, 275–285, doi:10.1016/j.ufug.2018.08.008.
68. De Nazelle, A.; Seto, E.; Donaire-Gonzalez, D.; Mendez, M.; Matamala, J.; Nieuwenhuijsen, M.J.; Jerrett, M. Improving estimates of air pollution exposure through ubiquitous sensing technologies. *Environ. Pollut.* **2013**, *176*, 92–99, doi:10.1016/j.envpol.2012.12.032.
69. Baig, M.M.; GholamHosseini, H.; Moqeem, A.A.; Mirza, F.; Linden, M. A Systematic Review of Wearable Patient Monitoring Systems—Current Challenges and Opportunities for Clinical Adoption. *J. Med. Syst.* **2017**, *41*, 115, doi:10.1007/s10916-017-0760-1.
